# Disease Burden and Sexual Life in Patients With Multiple Sclerosis: A Phenomenological Study

**DOI:** 10.1111/phn.70012

**Published:** 2025-08-17

**Authors:** Seher Çakmak, Sevda Uzun, Sevgi Demir Çam

**Affiliations:** ^1^ Department of Internal Medicine Nursing Faculty of Health Sciences Gümüşhane University Gümüşhane Turkiye; ^2^ Department of Psychiatric Nursing Faculty of Health Sciences Gümüşhane University Gümüşhane Turkiye; ^3^ Artvin Coruh University, Şavşat Vocational School Artvin Turkiye

**Keywords:** burden of disease, multiple sclerosis, qualitative study, sexual life

## Abstract

This study aims to examine the burden of disease experienced by multiple sclerosis patients and to deeply examine the impact of the disease on sexual life. A phenomenological research design was used in this study. This qualitative research was conducted with 20 multiple sclerosis patients. Interviews were conducted individually and face‐to‐face. The data were analyzed using Colaizzi's phenomenological analysis method. Two main themes (effects of the disease and sexual life) and five sub‐themes (physical effects, mental effects, social effects, problems related to sexual life, sexual issues, and coping) were identified in the analysis of the data. As a result of the study, it was determined that the patients experienced serious physical, mental, and social problems due to multiple sclerosis, and their sexual lives were also negatively affected. Individuals experience significant issues related to their sexual life due to multiple sclerosis. Since multiple sclerosis is a multifaceted disease, it is thought that professional evaluation of sexuality in patients, diagnosis of sexual dysfunctions, and taking timely measures are of great importance to improve the quality of life of multiple sclerosis patients.

## 1 Introduction

Multiple sclerosis (MS) is a chronic and progressive neurological disease characterized by demyelination of the central nervous system (CNS) that is common in young adulthood. Affecting sensory and autonomic functions, the disease is a serious public health problem that can lead to severe, irreversible disabilities (Kister et al. 2021).

People with MS have many and varying symptoms. These include physical symptoms such as stiffness in the hands and legs, balance problems, crooked lips, blurred vision, central visual halos, double vision, uncontrollable tremors, inability to lift objects, and the negative consequences of pregnancy; and psychosocial symptoms such as depression, anxiety, hostility, negative self‐concept, somatization, anger, fatigue, pessimism, and low self‐esteem (Pourhaji et al. 2023; Öz and Öz 2019; Marrie et al. 2015). In one study, the most common symptoms reported by MS patients were bowel and bladder dysfunction, fatigue, sleep disturbances, spasticity, and gait disturbance (Gustavsen et al. 2021).

Symptoms such as fatigue, depression, and spasticity, which are common in MS patients, cause disease burden (Barin et al. 2018). The prevalence of symptoms increases in people with MS with higher levels of disability. Fatigue, pain, and sleep disturbance have been shown to have the strongest associations with overall health. In addition, high rates of loss of bowel or bladder function, or both, have been reported in wheelchair‐bound and bedridden patients (Gustavsen et al. 2021). However, it is difficult to estimate the disease burden as some of these symptoms may be caused by other disorders and are common in the general population (Gustavsen et al. 2021). Disability and symptoms that develop due to the disease and exacerbate each other limit the functionality and activities of individuals' daily living and reduce their quality of life (Gustavsen et al. 2021; Newsome et al. 2022). Studies have reported that symptoms of walking difficulties, depression, pain, fatigue, tremors, anxiety disorders, and sexual dysfunction have a strong relationship with the quality of life in MS patients (Barin et al. 2018; Zhang et al. 2021).

Sexual dysfunction (SD) is a common multifactorial problem among people with MS. It can occur at any stage of the disease and with any level of disability (Ashtari et al. 2014). In a study of 2062 MS patients from 54 countries, more than half of the patients reported one or more problems with sexual function (Marck et al. 2016). In the literature, it is reported that female MS patients have higher rates of sexual dysfunction than male patients (Çelik et al. 2013). Among sexual dysfunctions, decreased libido is seen at similar and high rates in both sexes. Still, lack of sexual interest, difficulty in sexual arousal, and difficulties in reaching orgasm are more common in women. In contrast, erectile dysfunction, incomplete erections, erections that are not effective for penetration, and ejaculation failure are more common in men (Marck et al. 2016; Çelik et al. 2013; Kisic‐Tepavcevic et al. 2015). Duration of illness, physical competence, depression, fatigue, and lifestyle factors affect the frequency of having sexual intercourse and satisfaction with sexual function or sexual intercourse (Marck et al. 2016; Kisic‐Tepavcevic et al. 2015; Merghati‐Khoei et al. 2013).

There are three forms of sexual dysfunction in MS patients. Primary SD is a direct impairment of sexual responses or feelings due to neurological damage to the central nervous system. In primary SD, the most common complaints are lack of sexual interest, less intense orgasms, and inadequate vaginal lubrication/difficulty in achieving or maintaining a satisfactory erection. Secondary SD develops due to the burden of MS‐related symptoms. These symptoms often manifest as fatigue, weakness, spasticity, pain‐burning, memory‐concentration problems, bladder/bowel symptoms, and side effects of MS medications. Secondary SD is standard in both men and women, although women are more likely to suffer from bladder/bowel problems than men (Çelik et al. 2013). Tertiary SD is the shaping of sexual feelings and sexual responses due to psychological, emotional, or cultural influences of life. In tertiary SD, there is often a lack of trust, fear of sexual rejection, and anxiety about not being able to satisfy their partners (Guo et al. 2012). One study reported that fatigue was the strongest predictive factor for tertiary SD (Ashtari et al. 2014).

In Turkey, people with MS avoid talking to health professionals about the problems caused by the disease, especially about their sexual life, which is an essential part of these problems. These patients think that it is shameful to talk about sexual issues and hide their sexual problems. From this perspective, it is believed that this study will shed light on the sexual problems experienced by people with MS and will enable healthcare professionals to help guide those affected. This study aimed to examine the burden of disease experienced by MS patients and to examine the impact of the disease on sexual life deeply.

## 2 Materials and Methods

### 2.1 Study Design

Multiple sclerosis patients experience different psychosocial problems due to their disease burden. A phenomenological research design, which is a qualitative research design, was used to examine the disease burden experienced by multiple sclerosis patients and to examine the impact of the disease on sexual life in depth. This design was chosen because it offers a high degree of freedom in describing a phenomenon (event or experience) from the participants' perspective, providing rich data and detailing their experiences (Yıldırım and Şimşek 2016). This approach is critical in revealing the phenomenon's essence under investigation (Willig 2013; Morrow et al. 2015).

### 2.2 Working Group

The study was conducted in a public hospital in a province in southern Turkey between February 15 and May 15, 2024.

The criterion sampling method, one of the purposeful sampling methods, was used to determine the research study group. Criterion sampling is creating a sample from people, events, objects, or situations with the qualities determined for the problem (Altunay et al. 2014). The study sample consisted of MS patients who had applied to a state hospital and met the inclusion criteria. Inclusion criteria included being on MS treatment for at least 5 years and voluntarily participating; exclusion criteria included not accepting participation in the research and being on MS treatment for less than 5 years. All patients who met the inclusion criteria were invited to the neurology outpatient clinic in Adana City Hospital in the southern region of the country. The sample size was determined according to data saturation (Yıldırım and Şimşek 2016), and 20 participants were interviewed. When the study data started to repeat, the interviews were terminated based on data saturation. The study was also reported by the Consolidated Criteria for Reporting Qualitative Research (COREQ) guidelines (Tong et al. 2007) (Table 1).

**TABLE 1:** Combined criteria for reporting qualitative research (COREQ).

Number	Item	Guiding questions	Explanations
**Area 1: Research team and reflexivity**			
Personal characteristics			
1	Interviewer/facilitator	Which author/authors conducted the interview or focus group?	The third author conducted the interview
2	Identity information	What were the credentials of the researcher, e.g., PhD, MD	All authors have PhD
3	Profession	What was their occupation at the time of the study?	First author, Dr. Internal Medicine Nursing Second author, Dr. Psychiatric Nursing The third author, Dr. Internal Medicine Nursing
4	Gender	Was the researcher a man or a woman?	Three researchers are women
5	Experience and training	What experience or training did the researcher have?	The first author has completed qualitative research courses, possesses extensive experience in qualitative research. The second author has taken qualitative courses, has experience in qualitative research, and has published qualitative studies in international journals. The third author has completed qualitative research courses, possesses extensive experience in qualitative research.
Relationship with participants			
6	Relationship status	Was a relationship established before the training started?	No relationship was established before the start of the study
7	Participant's interviewer information	What did participants know about the researcher, e.g., personal goals, reasons for doing research?	Patients knew that the researcher had a PhD in internal medicine
8	Interviewer characteristics	What characteristics were reported about the interviewer/facilitator, e.g., bias, assumptions, motives, and interests in the research?	At the beginning of each interview, patients were informed about the aim and objectives of the study
**Area 2. Study design**			
Theoretical framework			
9	Methodological Orientation and Theory	Which methodological orientation was specified to support the study, e.g., discourse analysis, ethnography, phenomenology, content analysis?	This was a phenomenological study
Participant selection			
10	Sampling	How were participants selected? e.g., purposeful, convenience, consecutive, snowball	Criterion sampling method, one of the purposive sampling methods, was used
11	Approach method	How were participants approached? For example, face‐to‐face, telephone, mail	Face‐to‐face interviews were conducted with the patients before starting the study. The timing of the interviews was determined by the patients who voluntarily agreed to participate in the study
12	Sample size	How many participants were there in the study?	A total of 20 patients were included in the study
13	Disagree	How many people refused to participate or dropped out? Reasons?	No patient refused to participate in the study
Setting			
14	The setting of data collection	Where was the data collected? E.g., home, clinic, workplace	Detailed information is provided in the data collection section of the study
15	Presence of non‐participants	Was there anyone else present apart from the participants and the researchers?	Apart from the researchers, a nurse working in the hospital where the research was conducted was an observer
16	Description of the sample	What are the essential characteristics of the sample, e.g., demographic data, history	Patients who agreed to participate in the study and had been treated for multiple sclerosis for at least five years were included
Data collection			
17	Interview guide	Did the authors provide questions, prompts, and guidelines? Has it been pilot‐tested?	Detailed information was given in the Methods section
18	Repeat interviews	Have there been re‐interviews? If yes, how many?	No.
19	Audio/visual recording	Was audio or visual recording used to collect data in the study?	Interviews were recorded with a voice recorder
20	Field notes	Were field notes taken during and/or after the interview or focus group?	All patients' responses and researcher observations were recorded
21	Duration	How long were the interviews or focus groups?	Each interview lasted between 35 and 45 min
22	Data saturation	Has data saturation been discussed?	Data saturation was discussed
23	Transcripts returned	Have transcripts been returned to participants for comments and/or corrections?	No
**Area 3: Analysis and findings**			
24	Number of data coders	How many data coders coded the data?	Researcher 2 conducted the initial data coding, while researcher 1 and 3 reviewed the codes and provided revisions and feedback.
25	Description of the coding tree	Did the authors describe the coding tree?	The titles and subtitles in the results section represent the final coding tree
26	Derivation of themes	Were the themes predetermined or derived from the data?	Themes were derived from the data
27	Software	What software, if any, was used to manage the data?	Data were analyzed manually
28	Participant control	Provide feedback on the findings.	No
Reporting			
29	Quotes provided	Are participant quotes presented to illustrate themes/findings? Is each quote identified, e.g., participant number	Conclusion. Participant quotes are presented to illustrate themes/findings
30	Data and findings consistent	Was there consistency between the data presented and the findings?	Yes
31	Clarity of main themes	Are the main themes presented in the findings?	Yes
32	Clarity of small themes	Is there an explanation of the different cases or a discussion of minor issues?	Yes

### 2.3 Data Collection Tools

MS patients who came to the neurology outpatient clinic of a state hospital were interviewed. An in‐depth individual interview method was used in the study. In the interviews with the patients, a voice recorder was used in a suitable environment (bright, sufficiently lit, and ventilated). The interviews were conducted using a semi‐structured interview form prepared by the qualitative research method, which was created by the researchers based on the literature and revised in line with the opinions of five experts. Four main questions and sub‐questions related to disease burden and symptom management were used in the interviews.

1. In your opinion, what does it mean to have multiple sclerosis? What does a person with multiple sclerosis experience?
2. What are the problems you experience because of your illness? What do you do to cope with these problems? What methods do you use? What are your experiences?
3. What are the burdens of your illness? What are your experiences?
4. Is your sex life affected by your illness? What problems do you experience, and how does this affect you and your partner? What do you do to cope?

Preliminary interviews were conducted with two patients to test the questions' clarity and suitability for the study and to experience the interview process with the researcher. In the 35‐min preliminary interview, the patient's data were not included in the analysis. The duration of the interviews in the study varied according to the patient's statements but lasted between 45 and 55 min.

### 2.4 Data Analysis

In the analysis of the qualitative data obtained from the interviews, the 7‐stage analysis method developed by Colaizzi (1978) for phenomenological studies was used (Morrow et al. 2015). In this context, three researchers first read the interview texts independently and repeatedly. The data were tried to be understood, and the critical statements in the interview texts were selected, reorganized, and expressed in general terms. The researchers formulated and validated the meanings by discussing them until they reached a consensus. The researchers then identified and organized the themes into main and sub‐themes. The themes and sub‐themes of the study were developed through clear articulation. The accuracy of the themes and content was strengthened by presenting the survey findings to the participants. In addition, participants' statements were included so that the reader could verify the interpretation and analysis of the data.

### 2.5 Study Rigor

It is essential to ensure rigor and reliability in a qualitative study. This study's reliability is based on four criteria: credibility, dependency, verifiability, and transferability (Cypress 2017). For credibility, all participants were verbally informed about the purpose and study procedure and used an information sheet before inclusion. All participants were asked to sign a consent form. Interviews were conducted at a time and place convenient for both the interviewer and the interviewees. The interviews were conducted in a quiet room in the hospital's outpatient clinic, where the patient's privacy was protected. The third researcher had basic knowledge about the conduct of the interview and data collection. The interview tool was piloted before being applied to the interview. All interviews were recorded digitally and transcribed on the interview day. For reliability, the transcripts were re‐read to correct all possible errors. Three independent coders read all the transcripts, determined the codes separately, and discussed the differences. All researchers discussed and verified the identified codes, themes, and sub‐themes for verifiability. The transcription was done using purposive sampling, and the interviews were continued until data saturation was reached.

Consent to conduct the interview was obtained from the responsible physician in the neurology department at the selected Adana City Hospital. All participants were informed about the purpose and objectives of the study, possible benefits or harms, and their right to participate in the survey using the information form. Those who met the study criteria were asked to sign the consent form. They were informed that participation in the study was completely voluntary and that they could terminate their participation at any stage. The names of the participants were kept confidential, and all information was stored confidentially (Saunders et al. 2018).

### 2.6 The Research Team and Reflexivity

All three research team members are active faculty members in nursing schools. Two researchers have a PhD in internal medicine nursing, and one has a PhD in psychiatric health nursing. Two researchers have experience working as hospital clinical nurses and have received training in qualitative research methods. All three researchers have qualitative interviewing experience and have qualitative studies in international journals.

### 2.7 Ethical Aspects of the Research

This study was approved by the Gümüşhane University Scientific Research and Publication Ethics Committee (E‐95674917‐108.99‐223278). Written and verbal informed consent was obtained from the participants before starting the interview. Recordings and transcripts were stored on a password‐protected device. The study was conducted by the 1964 Declaration of Helsinki and the ethical standards of the National Research Committee.

## 3 Results

All participants were married, and 15 of them were women. The mean age of the individuals included in the study was 36.75 ± 8.8 years. Three of the individuals had an additional disease other than MS. Demographic and occupational characteristics of the individuals who participated in the study are presented in Table 2. Participants are not in remission but are in the relapse phase. In addition, participants do not show common MS symptoms such as bladder/bowel dysfunction or impaired mobility. Some participants receive psychological support, and two participants are taking psychiatric medication (antidepressants).

**TABLE 2:** Characteristics of the participants.

Participant number	Age	Gender	Marital status	Education status	How many years has he had MS	An additional disease	Use of medication that affects sexuality
P1	29	Woman	Married	License	5 years	No	No
P2	44	Male	Married	High School	15 years	Diabetes	No
P3	32	Male	Married	Primary School	10 years	No	No
P4	53	Male	Married	Primary School	18 years	No	No
P5	42	Woman	Married	Primary School	6 years	No	No
P6	29	Woman	Married	Middle School	5 years	No	No
P7	41	Male	Married	Primary School	12 years	No	No
P8	30	Male	Married	License	8 years	Epilepsy	No
P9	40	Woman	Married	High School	10 years	No	No
P10	56	Woman	Married	Primary School	20 years	Type 2 diabetes	No
P11	29	Woman	Married	High School	5 years	No	No
P12	29	Woman	Married	High School	5 years	No	Yes
P13	28	Woman	Married	Primary School	5 years	No	Yes
P14	48	Woman	Married	Primary School	10 years	No	No
P15	37	Woman	Married	High School	5 years	No	No
P16	29	Woman	Married	High School	5 years	No	No
P17	36	Woman	Married	High School	5 years	No	No
P18	44	Woman	Married	University	9 years	No	No
P19	33	Woman	Married	Primary School	5 years	No	No
P20	26	Woman	Married	Primary School	5 years	No	No

As a result of the data analysis obtained from the semi‐structured interviews, themes, sub‐themes, and codes were identified (Table 3).

**TABLE 3:** The burden of disease and sexual life in multiple sclerosis.

Themes	Sub Themes	Codes
1. Effects of the disease	A. Physical effects	A1. Pain in the legs A2. Problems with balance A3. Muscle pain A4. Disruption in sleep pattern A5. Difficulty walking A6. Loss of vision A7. Numbness in the hands A8. Fatigue A9. Speech impairment A10. Double and blurred vision A11. Urinary incontinence A12. General body tremor A13. General body pain A14. Coordination disorder A15. Tingling in hands and arms A16. Quitting the job A17. Powerlessness A18. Constipation A19. Paralysis
	B. Mental effects	B1. Decreased self‐confidence B2. Anxiety B3. Depression B4. Sadness B5. Despair B6. Burnout B7. Nervousness B8. Anger B9. Unhappiness B10. Inability to enjoy life
	C. Social impacts	C1. Decreased social activity C2. Decreased quality of life C3. The desire to emotionally distance from people C4. Withdrawal from society
2. Sexual life	A. Problems with sexual life	A1. Sexual aversion A2. Fatigue A3. Pain during sexual intercourse A4. Sexual dissatisfaction A5. Wanting to get pregnant but not being able to and consequent sexual reluctance A6. Anxiety and fear of having sexual intercourse A7. Thinking that the spouse is estranged from them A8. Not wanting to share the same bed with their spouse A9. Thinking about leaving your spouse A10. Inability to orgasm A11. Urinary incontinence during sexual intercourse
	B. Sexual problems and coping	B1. Not coping and thinking about divorce B2. Getting psychological support B3. To hold on to life with the support of your spouse B4. Trying to feel spiritually strong B5. Receiving social support B6. Praying B7. Taking vitamin supplements B8. Consuming blueberries

### 3.1 Theme 1. Effects of Disease

#### 3.1.1 Subtheme 1. Physical Effects

As a result of the interviews with patients, it was determined that MS disease has effects such as pain, problems with balance, muscle pain, disruption in sleep patterns, difficulty walking, loss of vision, numbness in the hands, fatigue, impaired speech, double and blurred vision, and incontinence.

“I have decreased visual acuity, insomnia at night, urinary incontinence, vision problems, I constantly go to check‐ups, my eyes were affected during the attack period, and the problem continues, this situation affected me very badly.” (P8)

“I have vision loss in my right eye. I have had multiple sclerosis for 2 years; insomnia happens occasionally, now I come to the doctor for regular check‐ups, I take my medication, I see serious side effects if I miss my medication, I pay attention to my diet.” (P12)

“I have slowed down movement in my legs and arms; I was diagnosed in 2012, my face was semi‐paralyzed, I have trouble walking when I can't walk, I sit, lie down, and don't move…It is a difficult disease…” (P19).

#### 3.1.2 Subtheme 2. Mental Effects

According to the data obtained from the interviews with the patients, it was stated that patients with multiple sclerosis were negatively affected mentally. It was determined that feelings of fear, anxiety, depression, sadness, and helplessness were experienced.

“Normally, I try to be calm, but when someone touches me, I get furious; this has started to interfere with my life. I don't think I can cope; I am furious, and I don't know what to do.” (P11).

“I am furious; I started taking psychiatric drugs, I go to a psychiatrist, I try to calm down, but I am not very successful. Sometimes, I reflect on this with my family; it is a problematic disease…Even though I try to cope with it, I find it difficult…” (P13).

“I have visual impairment, numbness in my hands, fatigue. My diet has deteriorated, I have lost weight in the last 6 months, there is numbness in my face, and I cannot find anything to deal with; I come to the doctor when my complaint increases a lot. But it affects me very negatively mentally. It is a bad disease; it neither cures nor kills…” (P4).

#### 3.1.3 Subtheme 3. Social Impacts

According to the data obtained from the interviews, it was determined that the treatment of multiple sclerosis had a negative social impact on the patients.

“Numbness in the feet, legs, and hands, blurred eyes, dizziness, I cannot do anything, I withdraw myself in everything, numbness in the feet, I have difficulty standing and walking, it hits my tongue, sometimes I have difficulty speaking.” (P5).

“You have no social life; you have difficulty walking. You withdraw yourself from society. You are already unhappy, you don't enjoy anything…When that happens, you don't feel like doing activities…” (P20)

### 3.2 Theme 2. Sexual Life

#### 3.2.1 Subtheme 1. Problems Related to Sexual Life

According to the data obtained from the interviews, patients experience various difficulties during sexual activity. Patients reported experiencing sexual reluctance, sexual dissatisfaction, and pain during sexual intercourse.

“I have four children; I feel depressed, I feel restless, my legs and muscles ache a lot, I have no sexual desire, my husband does not force me, I am already depressed in general.” (P15)

“Sexual life is negatively affected, my sexual life is over, I have problems with my spouse, it is horrible to be sick, I have issues with my spouse, we cannot overcome them, I am thinking of divorce, I cannot even sleep in the same bed, I have urinary problems, and my pain is tough for me. I leak urine during sexual intercourse, and this reflects on my husband; he is uncomfortable, and he has no desire for sexual intercourse; it is a terrible situation.” (P7)

#### 3.2.2 Subtheme 2. Sexual Problems and Coping

According to the data obtained from the interviews with the patients, the patients stated that they experienced problems due to multiple sclerosis and used methods such as not being able to cope and thinking about divorce, receiving psychological support, holding on to life with the support of their spouse, trying to feel spiritually strong, receiving social support, praying, taking vitamin support and consuming blueberries to cope with these problems.

“I experience sexual weakness and fatigue a lot. I don't want sexual intercourse. I have problems with my husband, and I force myself to have intercourse, but it doesn't work; this is a big problem for us. I pray to God to cure this disease.” (16).

“My sexual life is affected; being tired all the time, having dizziness, it affects me a lot; I don't want to sleep with my husband; I get away from him. I force myself to have sexual intercourse. I want to get rid of this disease; I want to make my husband happy…I sometimes take vitamin support, and I hear it is good…” (P12)

“I can't be with my husband; I don't have the will and strength, and my severe dizziness doesn't even allow me to sleep or lay my head on the bed. There is no pleasure in sexual life anyway. I pray to God, and my husband tries to support me. I love him very much, but I know that sexual life is significant…” (P10)

## 4 Discussion

This study aimed to examine the burden of disease experienced by MS patients and to examine the impact of the disease on sexual life in depth. The burden of disease and sexual life in patients with multiple sclerosis were analyzed in two categories.

In this study, it was found that individuals with MS were negatively affected in physical, mental, and social aspects. In our study, MS patients reported experiencing physical effects such as pain, problems with balance, muscle pain, disruption in sleep patterns, difficulty walking, vision loss, numbness in hands, fatigue, speech impairment, double and blurred vision, and incontinence; mental effects such as fear, anxiety, depression, sadness, and helplessness; and social effects such as withdrawal from society and reluctance to do activities. Other studies in the literature also support our study. Hosseini et al. (2022) reported that MS patients experienced physical disorders such as sensory‐motor problems, vision problems, speech and swallowing issues; cognitive disorders, bladder dysfunction, excessive fatigue, unstable body temperature, balance and gait disorders, impotence and sexual dysfunctions; psychological problems such as depression, stress, hopelessness, anxiety, bipolar disorder, personality disorders, loneliness, stressful thinking habits and emotional fatigue; and social disorders such as social exclusion and decreased social and interpersonal interaction (Hosseini et al. 2022). Yılmaz et al. (2017) found in their study on women with MS that women had at least one of the symptoms, including numbness in the extremities, loss of sensation, aphasia, balance or vision complaints, and that women were concerned about the effects of MS on their physical health and the progression of MS (Yılmaz et al. 2017). Gustavsen et al. (2021) reported that MS patients frequently experience symptoms of bowel and bladder dysfunction, fatigue, sleep disturbances, spasticity, and gait disturbances (Gustavsen et al. 2021). Rommer et al. (2019) found that the most frequently reported symptoms by MS patients were fatigue, spasticity, and voiding disorders (Rommer et al. 2019). In our study and similarly in other studies, it is seen that the symptoms associated with MS are very diverse. Multiple symptoms can seriously affect the activities of daily living and quality of life of individuals with MS. Patients face physical, emotional, psychological, and behavioral changes, fear disability, endure the financial burden of the disease, and confront cultural and social misconceptions (Dehghani et al. 2019). Therefore, the characteristics of the symptoms that develop in MS patients should be questioned in detail, especially by nurses, and the necessary physical or psychosocial support should be provided.

In our study, people with MS reported experiencing sexual reluctance, sexual dissatisfaction, and pain during sexual intercourse. They found it challenging to cope and used a variety of coping strategies. These strategies include spirituality, partner support, and supplements. In a study conducted by Esteve‐Ríos et al. (2022) to investigate the sexual experiences of women with MS, it was reported that the main factors affecting women's sexual relationships include orgasmic changes such as lubrication problems, shortened orgasm duration or difficulties in reaching orgasm or lack of libido, all of which have the potential to cause these women to feel rejection or insecurity in their relationships. In the same study, their willingness to improve their sexual health by actively seeking resources that can help improve their partner's understanding and sexual expression is essential to them (Esteve‐Ríos et al. 2022). Nazari et al. (2020) found that 69.8% of women with MS had general sexual dysfunction, and approximately two out of five had problems with sexual desire, sexual arousal, and orgasm (Nazari et al. 2020). In a study conducted by Yılmaz et al. (2017) in women with MS, it was found that women had decreased number of intercourse, avoidance of intercourse, loss of sexual appetite, lack of arousal due to insensitivity, lack of orgasm, and decreased sexual satisfaction. In the same study, it was reported that some of the husbands of the women acted understandingly by trying to support their spouses due to the changes that occurred in the lives of women with MS.

In contrast, others did not make any complaints or behaved more humiliatingly (Yılmaz et al. 2017). Similar to this study, other studies have shown that people with MS experience severe sexual problems as a result of the disease and develop coping mechanisms such as understanding and support from their partners. The adverse effects of a disease such as MS on sexuality may cause individuals to feel inadequate, question themselves, and have serious concerns about pleasing their spouses. Individuals with MS who cannot receive support from their spouses in sexuality consider divorce or resort to different ways to cope with this situation. Nurses should encourage these patients to talk about sexuality and support them to express their concerns easily. When assessing sexual functions in MS patients, nurses are advised to ask questions such as how MS affects the patient's sexuality and the way they see themselves as partners, how crucial sexual intimacy is, how often they engage in sexual activities, whether this is normal/unusual for them, whether they are satisfied with their sexual responses, and what information, interventions, or resources they would like to help them perform their sexual functions (Fletcher et al. 2009).

## 5 Conclusion

As a result, it was determined that the physical, mental, and social effects of the disease constitute a significant burden on patients with MS. It was also seen that this condition leads to the development of various disorders in sexual functions, which are a fundamental aspect of life. It was determined that patients resort to different methods of coping with sexual dysfunctions. Therefore, the burden and sexual problems related to the disease are essential issues to consider in the treatment process. Nurses should encourage MS patients to seek support for sexual dysfunction.

### 5.1 Limitations

One of the limitations of the study is that all participants were from the neurology outpatient clinic of a state hospital in a city in southern Turkey. The results depend on the participants and the setting in which the study was conducted. The small group of participants is not representative of the MS patient population.

## Conflicts of Interest

The authors declare no conflicts of interest.

## Data Availability

Data sharing is not applicable to this article as no new data were created or analyzed in this study.
